# Cumulative lifetime violence severity, social determinants and anxiety in a national sample of Canadian men

**DOI:** 10.1186/s12888-022-03865-8

**Published:** 2022-04-14

**Authors:** Kelly Scott-Storey, Sue O’Donnell, David Busolo, Enrico DiTommaso, Jeannie Malcolm, Petrea Taylor, Charlene D. Vincent, Judith Wuest

**Affiliations:** 1 Faculty of Nursing, University of New Brunswick, P.O. Box 4400, Fredericton, NB E3B 5A3 Canada; 2 Faculty of Nursing, University of New Brunswick, Moncton, Canada; 3 Department of Psychology, University of New Brunswick, Saint John, Canada

**Keywords:** Anxiety, Cumulative lifetime violence, Men, Gender, Socio-economic disparity, Social determinants

## Abstract

**Background:**

Despite anxiety disorders being the ninth leading cause of disability and associated with social inequities, little attention has been given to how intersections among social determinants of health and chronic stressors such as cumulative lifetime violence affect the likelihood of experiencing anxiety disorders. Our purpose was to explore the relationships among cumulative lifetime violence severity as target and perpetrator, social determinants of health and generalized anxiety disorder in Canadian men.

**Methods:**

Using a community sample of 592 Canadian men who self-identified as having experienced violence, we developed and tested an evidence-based model of generalized anxiety disorder including indicators of cumulative lifetime violence, gender, social location, socio-economic disparity, personal resources and other chronic stressors using logistic regression.

**Results:**

Most men (76.4%, *n* = 452) reported experiences as both target and perpetrator. The model accounted for 50.8% of the variance in anxiety severity χ^2^ (8) = 264.43, *p* = .000). The prevalence of probable generalized anxiety disorder was 30.9%, a rate higher than that found among Canadian men in general in the same period. Remarkably, the likelihood of generalized anxiety disorder increased by a factor of 5.30 for each increase of 1 in cumulative lifetime violence severity, and six-fold for feeling overwhelmed by demands of everyday life (*a*OR = 6.26). Masculine discrepancy stress, having been born in Canada, unemployment, and food insecurity also contributed significantly to increasing the likelihood of generalized anxiety disorder. Both social support and mastery had significant aORs < 1, suggesting possible protective effects. Together these findings delineate characteristics and social determinants that may heighten vulnerability to generalized anxiety disorder and influence its progression among men who have experienced lifetime violence.

**Conclusions:**

These findings are the first evidence that Canadian men with lifetime violence histories are a sub-group disproportionately affected by chronic stressors and socio-economic disparities and that together the presence and/or severity of these factors increases their vulnerability to generalized anxiety disorder. Our results highlight the importance of strengths-based trauma- and violence-informed approaches to care, including practical resources to reduce the stress of everyday life, improve social support, and reinforce personal control and choice.

## Background

In the study of anxiety, limited attention has been given to people disproportionately affected by social inequities and/or chronic stressors such as interpersonal violence [1]. Globally, anxiety disorders are the ninth leading cause of years lived with disability [2]. Anxiety disorders frequently are more impairing than many chronic physical disorders; yet first treatment often does not occur until more than a decade after symptom onset [3]. Social determinants of health (SDOH) such as sex, gender, age, immigration status, coping resources, and socio-economic factors have been associated with the experience and course of generalized anxiety disorder (GAD) [4–6]. Knowledge of how intersections among SDOH and chronic stressors increase or decrease likelihood of experiencing GAD is important for identifying more vulnerable groups and developing tailored prevention and treatment strategies.

### Cumulative lifetime violence and anxiety

One such group is men with histories of violence. Men’s experiences of violence as target and/or perpetrator are pervasive across the lifespan [7]. Toxic stress from chronic, unpredictable, recurrent violence as target or perpetrator dysregulates the acute stress response and leads to allostatic overload, neurobiological changes, and chronic physical and mental health problems [8]. Among men, the 12-month prevalence rate of anxiety disorders (14.3%) *exceeds* that of disorders of mood (7.7%), impulse control (7.7%), alcohol (4.5%) and drugs (2.2%), suggesting that anxiety is a considerable mental health problem for some men [5, 9]. Discrete experiences of violence are associated with anxiety among men; for example, child maltreatment [10, 11]; intimate partner violence (IPV) [12, 13]; sexual violence victimization [14]; childhood bullying [15]; workplace violence [16]; gang violence perpetration [17]; and gender-based violence perpetration [18]. A limitation of this body of research is a focus on the associations between anxiety and only one or two types of violence in one or two contexts without considering the potential *cumulative* and overlapping effects of other lifetime violence exposure [19].

To overcome this shortcoming, we recognized the need to study cumulative lifetime violence which we defined as perceived physical, psychological and sexual violence or abuse experiences in childhood (under 18 years) and adulthood, as target and/or perpetrator, in diverse contexts including families, partner relationships, schools, communities, and workplaces [20]. We developed a measure of cumulative lifetime violence severity (CLVS) and found CLVS to be significantly associated with anxiety severity (*r* = 0.477, *p* < 0.001) in a convenience sample of 685 men [20]. To our knowledge, our findings provided the first evidence that CLVS is a factor that may identify men more vulnerable to GAD. Notably, our findings were limited to eastern Canadian men and did not consider factors that may exacerbate or reduce anxiety among men with lifetime violence histories. Violence of all types has been strongly associated with SDOH [21] supporting the need to explore how SDOH-related inequities may interconnect with cumulative lifetime violence (CLV) to increase the likelihood of GAD among men.

### Social determinants of health and anxiety

In the present study of men, gender is a central SDOH. Men construct and express gender in terms of socially prescribed masculine roles, values and behaviors [22]. *Gender role discrepancy stress* is experienced by men who are disturbed by their perception that they are less masculine than the ‘typical’ man [23]. *Discrepancy stress* may initiate efforts to validate masculinity to self and others through risky behaviors with potentially negative health consequences, for example, substance use, sexual behavior, and violence perpetration among adolescent boys [24]. Qualitative findings show that anxiety is experienced by some men as a loss of control, characterized by a sense of failure and powerlessness that contrasts with dominant social norms of masculinity that value strength, emotional control, and self-reliance [25]. This discovery is consistent with the theory that personal resources such as perceived mastery and/or social support may buffer or exacerbate the relationship between a stressor and mental health outcomes [26]. Among men, perceived mastery or personal control was found to partly mediate the relationship between psychological and physical IPV as target and comorbid anxiety and depressive symptoms among men but social support did not [27]. Yet, using interview data, Taylor et al. [28] found the main mental health manifestation of CLV among men with anxiety, depression and/or posttraumatic stress disorder (PTSD) was perceived detachment from others fueled by perceptions of gender role expectations to be independent, stoic and self-reliant. Men were found to rectify detachment by attempting to gain self-worth in relationships by *connecting* with others, an approach that contradicts the prevailing demand to solve problems independently like a “man”. These findings suggest that the associations among mastery, perceived social support and anxiety may be complex in the context of gender and CLV.

Additionally, although men are perceived to benefit from social and economic power differentials between men and women, not all benefit in the same way, and economic inequities among men may accentuate or buffer health effects of other SDOH [29]. Socio-economic disparities that have been associated with anxiety include: food insecurity [30, 31], adverse housing [32, 33], unemployment [34, 35], low income [36], and financial strain [37]. Mental health problems including anxiety have been linked to the stress of everyday life from material and social conditions worsened by organized violence in conflict situations [38].

Indicators of social location such as sexual orientation, ethnicity/culture, and age have also been associated with anxiety. Mood and anxiety disorders are more prevalent among gay and bisexual men than heterosexual men [39]. Cultural variation in the prevalence and course of mental health problems is linked to differences in interpretation of symptoms, historical trauma, or strengths of community support systems [40]. For example, compared to those born in Canada, immigrants are less likely to experience anxiety [6]. Anxiety disorders begin at an early age and have substantial subsequent morbidity and comorbidity [3]. In community samples, onset of chronic physical conditions such as arthritis, asthma, hypertension, chronic obstructive lung disease and chronic pain has been associated with anxiety disorders [41].

In summary, despite evidence to support significant associations between anxiety and individual SDOH in general populations including men, there is a paucity of knowledge about how CLV and SDOH together influence anxiety in men. To address this gap in knowledge about men with CLV, we developed a multivariable model of GAD that included indicators of CLV, gender (gender discrepancy stress), social location (age, immigration status, Indigeneity, sexual orientation), socio-economic disparity (employment, financial strain, food insecurity, adverse housing, personal income), personal resources (social support, mastery) and biophysical pathways from violence to anxiety (frequency overwhelmed by demands of everyday life, chronic physical conditions).

### Aims

Our aims in this exploratory analysis of anxiety in men reporting CLV are to: 1) develop a descriptive profile of and examine differences between CLV and SDOH by GAD levels; 2) test the hypothesis that GAD is associated with and predicted by CLV; and 3) test our *multivariable* model by examining how GAD is a) associated individually with each theoretical concept in the model, and b) predicted by these theoretical concepts.

## Methods

We received funding to expand our research program to a nationally representative sample of Canadian men with CLV, including additional measures of diverse SDOH associated with health disparities in January 2020. We received approval from the University of New Brunswick Research Ethics Board and engaged Qualtrics^©^ research panel services to recruit participants in March 2020 when research activities were interrupted by the COVID-19 pandemic; thus, data collection was delayed until June 2020. No specific questions related to COVID-19 were included in the survey.

A community convenience sample of English-speaking individuals, aged 19 years and older, living in any Canadian province or territory who self-identified as men with experiences of violence (physical, psychological and/or sexual) as a target and/or perpetrator in their lifetime were recruited using Qualtrics^©^ research panel services. Qualtrics^©^ maintains a large pool of respondents with diverse demographics who have agreed to be contacted to take part in surveys. Thus, response rates are higher than those of traditional recruiting methods and the sample is representative of the target group. To strengthen diversity, our sample was stratified by age groups and regions and included 20% born outside of Canada. Interested men were directed to an online screening page and those eligible linked to the letter of information. Informed consent was received online before the survey link was released. Upon completion, men were directed to a debriefing page to review signs and symptoms of possible distress related to survey completion and strategies and resources for managing that distress. Additionally, Qualtrics^©^ awarded each participant an incentive of reward points valued at approximately two Canadian dollars (CAD) and a $15 CAD gift card to acknowledge their time.

Recruitment resulted in 636 men completing the survey. Qualtrics^©^ prepared a scrub report on data quality, based on criteria such as speeding, flatlining, and duplication. We reviewed cases identified in this report and removed 35 cases due to dubious or poor data quality. In addition, 3 cases were removed due to excessive missing data (more than 30%) on the violence scale. Missing data was minimal (< 5%) and in scales missing 20% or less, data were replaced with mean case scores on the scale. In the current analysis, 6 cases were removed because each had excessive missing data on one key variable, resulting in a sample of 592.

### Measures

Self-report questions with categorical check boxes were used to collect socio-demographic information such as age, sexual orientation, geographic location, education, employment and income as well as chronic physical health problems. Established tools were used to measure CLV, gender discrepancy stress, some SDOH, and anxiety. We used the CLVS-44 scale as perpetrator and target in childhood and adulthood to collect data about the severity of men’s experiences of CLV [20]. For each of 44 items, men rated frequency, from 1 (never) to 4 (often), and degree of distress, from 1 (not at all) to 4 (very). Frequency and distress scores were summed and averaged for a severity score of 1 to 4 on each item, with higher scores indicating greater severity. Item severity scores were summed and averaged for a total CLVS-44 scale (range 1 to 4) and sub-scale scores (range 1 to 4) for each of 11 subscales. The CLVS-44 has excellent internal consistency (α = .92) and convergent validity of *r* = 0.75 (*p* < *.001*) with a global violence severity score (0 to 10). In the current analysis, the CLVS-44 total score α = .94. The subscale alphas were: C1–Lifetime Perpetrator Physical and Psychological Violence (not partner or work) (α= .86); C2–Childhood Target Physical and Psychological Peer/Team Violence (α= .81); C3–Lifetime Perpetrator Sexual Violence (α= .91); C4–Adult Target Psychological Violence—Workplace, Messaging, or Stalking (α= .72); C5–Childhood Target Sexual Violence (α= .76); C6– Adult Target and Perpetrator Violence Related to Nature of Work or Civil/Political Unrest (α = .81); C7–Lifetime Target Physical and Psychological Dating/Partner Violence (α = .79); C8–Lifetime Target Physical Violence from Family Members or Others with Power Over Them (α = .73); C9–Lifetime Perpetrator Stalking and Messaging (α = .84); C10–Adult Perpetrator Workplace Psychological and Gender-based (or Other Characteristic) Violence (α = .73); and C11–Lifetime Perpetrator Physical Dating/Partner Violence (α = .70).

To measure masculine discrepancy stress (MDS), we used the 5-item Discrepancy Stress subscale from the Gender Role Discrepancy and Discrepancy Stress Scale [23]. Men responded to 5 items on a scale from 1 (strongly disagree) to 7 (strongly agree) about how they feel about statements such as, “I wish I was more ‘manly,’” or “I worry that people judge me because I am not like the typical man”. Scores for items were summed for a score of 5 to 35 with higher scores reflecting greater MDS. Internal consistency was found to be .86 in foundational studies of MDS and high-risk sexual behaviour and of IPV perpetration [23, 42]. In this study, α = .90 for the MDS subscale.

Men were asked to rate a single question adapted from the Financial Strain Index, “Overall how difficult is it for you to live on your income right now?” on a scale from 1 (not at all difficult) to 4 (extremely difficult to impossible) [43]. Food insecurity in the past 12 months was assessed with a response of *often* or *sometimes* to at least one item on a 2-item screen asking how often they worried that food would run out before they had money to buy more, and how often the food they bought did not last and they did not have money to get more [44]. We assessed adverse housing in the past 12 months with a positive screen to at least one of the following: whether they had moved 2 or more times; whether there was a time when they were unable to pay rent or mortgage on time; and whether there was a time they did not have a steady place to sleep or slept in a shelter [45]. Stress from demands of everyday life was measured with a self-report question, “In a typical week, how often do you feel overwhelmed or stressed by the demands in your life?” with response options of *never or seldom*, *a few times*, *often* and *most of the time* which were dichotomized for analysis.

Perceived social support as a personal resource was measured with the Medical Outcomes Scale, Social Support Short Form [46]. Participants responded to 5 items about how often (*none of the time* to *all of the time*) emotional, informational, and instrumental assistance was available on a 5-point scale for a total summative score from 5 to 25, with higher scores representing greater perceived social support. The short form scale has been validated in a community sample of people with chronic illnesses and internal consistency was .88 [46]. In the current analysis, α = .87. We measured the extent to which people feel in control of their life situation with the 7-item mastery scale, shown to be unidimensional in confirmatory factor analysis [47]. For each statement about personal control, participants indicated agreement from 1 (strongly agree) to 4 (strongly disagree) for a summative score of 4 to 28, with higher scores indicating greater mastery. In a study of male veterans, α = .83 and in the present study α = .79.

We used the 7-item Generalized Anxiety Disorder scale (GAD-7) to identify *clinically* significant GAD as our model outcome. The GAD-7 measures frequency of anxiety symptoms over the previous two weeks on a 4-point scale (*not at all* to *nearly every day*); summative scores range from 0 to 21 [48]. Continuous scores were dichotomized to capture probable GAD (moderate to severe anxiety) with a score > 9 and no significant GAD (none to mild) with scores < 10 [48]. Reliability and construct validity have been established in the general population [49] and in our previous study of men and lifetime violence, α  = .94 [20]. In the present study, α  = .93.

### Analysis

We used IBM^©^ SPSS^©^ Version 27. Descriptive statistics were computed for all variables. Binary logistic regression (LR) was used to examine unadjusted odds ratios (OR) and to test multivariable models. Assumptions of independence, linearity, and lack of multicollinearity were met for each LR unless otherwise specified in results. To validate final multivariable models, we examined significance of predictors according to bias-corrected and accelerated (BCa) confidence intervals (CI). GAD severity was dichotomized as probable GAD by a GAD-7 score > 9 and no significant GAD by a GAD-7 score < 10. For aim 1, descriptive statistics and bivariate comparisons (t-test or χ^2^) for each variable in the model were calculated by GAD category. To achieve aim 2, we used LR to calculate ORs for GAD by the total CLVS-44 score and each of the 11 CLVS-44 subscale scores. Using simultaneous entry, we also examined adjusted odds ratios (*a*OR) in a model containing all CLVS-44 subscales as predictors of GAD. For aim 3, we examined each concept indicator in our model as an unadjusted predictor using LR. To avoid spurious associations or dilution of true associations or large standard errors with wide confidence intervals, we included only those indicators that were significant at < .1 [50] in the multivariable LR model. Specifically, CLV; gender; indicators of social location (age, immigration status, sexual orientation); indicators of socio-economic disparity (employment, financial strain, food insecurity, adverse housing, income); personal resources (social support, mastery/perceived control) and indicators of allostatic overload (frequency overwhelmed by demands in typical week, chronic physical conditions) were entered simultaneously as possible predictors of GAD. Significance for all tests was assessed with a *p* value less than .05. Our sample of 592 exceeded the minimum recommendation of 500 for LR [51].

## Results

### Description of sample

Of the 592 men, most (76.4%, *n* = 452) reported experiences of violence as both target and perpetrator; 23.3% (*n* = 139) as target only, and 0.2% (*n* = 1) as perpetrator only. Men ranged in age from 19 to 88 years (µ = 47.4, SD = 16.3) with 10.8% (*n* = 64) ages 19 to 24, 33.6% (*n* = 199) ages 25 to 44, 35.5% (*n* = 210) ages 45 to 64, and 20.1% (*n* = 119) 65 and older. Most identified as heterosexual (86.1%; *n* = 510) and 1.2% (*n* = 7) as transgender. Geographically, 13.5% (*n* = 80) lived in British Columbia, 17.4% (*n* = 103) in the Prairie provinces, 37% (*n* = 219) in Ontario, 23.8% (*n* = 141) in Quebec, 8.1% (*n* = 48) in Atlantic Canada, and 0.2% (*n* = 1) in the Northwest Territories. Additionally, 60.6% (*n* = 358) lived in large cities (< 100,000), 18.4% (*n* = 109) in cities of 30,000 to 99,999, 14.7% (*n* = 87) in small towns of 1,000 to 29,999, and 6.2% (*n* = 37) in rural communities of less than 1000.

The majority (79.6%; *n* = 471) were born in Canada and 20.4% (*n* = 121) were newcomers, most of whom (85.1%; *n* = 103) had lived in Canada for more than 5 years. Only 6.8% (*n* = 40) identified as Indigenous Canadian. About one quarter (23.6%, *n* = 140) had dependent children. For education, 59.6% (*n* = 353) had college diplomas or university degrees, 21.6% (*n* = 128) high school education or less and 18.8% (*n* = 111) some post-secondary education. The majority were employed (57.8%, *n* = 342); 22.3% (*n* = 132) received a retirement pension, 17.6% (*n* = 104) old age security pension, 17.1% (*n* = 101) employment insurance, 16.7% (*n* = 99) social assistance, and 9.0% (*n* = 53) disability pension. For annual income in CAD, 29.1% (*n* = 172) reported less than $25,000, 24.7% (*n* = 146) between $25,000 and $49,999; 17.1% (*n* = 101) between $50,000 and $74,999, and 29.2% (*n* = 173) more than $75,000. The mean score on the GAD-7 was 7.01 (SD = 5.76; range 0 to 21).

### Aim 1. CLV and SDOH by levels of gad

See Table 1 for a descriptive profile and differences between CLV and SDOH by *probable GAD* and *no significant GAD*. In comparison to the 409 (69.1%) men with no significant GAD, the 183 (30.9%) with probable GAD differed significantly; they were younger, with higher mean scores on CLV, masculine discrepancy stress, and number of chronic physical conditions and lower mean scores on mastery and social support. Additionally, a significantly higher percentage of those with probable GAD did not identify as heterosexual and were experiencing financial strain, food insecurity, and adverse housing; living on less than $50,000 CAD per year; and feeling overwhelmed by demands in a typical week often or most of the time. Although a greater proportion of men with probable GAD were likely to be born in Canada, unemployed, and Indigenous Canadian than those with no significant GAD, these differences were not statistically significant.

**Table 1:** Descriptive Profile of Generalized Anxiety Disorder (GAD) by Model Predictors (*N* = 592)

**Predictor Variables**	**Probable GAD**^a^ **(*n* = 183)**	**No Significant GAD**^a^ **(*n* = 409)**	**Tests of Differences between Groups**
	**μ (SD)**	**μ (SD)**	***t-Tests***
**CLVS** ^b^	1.79 (.56)	1.41 (.28)	-8.63, *p* < .001*
**Age in Years**	42.50 (15.75)	49.61 (16.1)	5.00, *p* < .001*
**Masculine Discrepancy Stress**	18.98 (8.60)	13.87 (7.39)	-6.97, *p* < .001*
**Social Support**	12.60 (5.12)	15.17 (5.24)	5.55, *p* < .001*
**Mastery**	16.24 (3.64)	19.53 (4.11)	9.77, *p* < .001*
**Number of Chronic Physical Conditions**	1.41 (1.54)	1.9 (1.94)	-3.35, *p* < .001*
	**n (%)**	**n (%)**	**χ**^**2**^
**Immigration Status**			
Born in Canada	154 (84.2)	317 (77.5)	3.44, *p* = *.06*
Newcomer	29 (15.8)	92 (22.5)	
**Indigeneity**			
Indigenous Canadian	13 (7.1)	27 (6.6)	0.05, *p* = *.82*
Not Indigenous Canadian	170 (92.9)	382 (88.0)	
**Sexual Identity**			
Heterosexual only	150 (82.0)	360 (88)	3.88, *p* = .05*
Gay, Bisexual, Another Sexual Identity	33 (18.0)	49 (12.0)	
**Current Employment**			
Employed	96 (52.5)	246 (60.1)	3.06, *p* = *.08*
Not Employed	87 (47.5)	163 (39.9)	
**Difficulty Living on Income**			
Not at all to a little	105 (57.4)	357 (87.3)	66.00, *p* < .001*
Difficult to impossible	78 (42.6)	52 (12.7)	
**Food Insecurity**			
Food Secure	55 (30.1)	305 (74.6)	105.14, *p* < .001*
Food Insecure	128 (69.9)	104 (25.4)	
**Adverse Housing**			
Stable Housing	102 (55.7)	356 (87.0)	70.75, *p* < .001*
Adverse Housing	81 (44.3)	53 (13.0)	
**Personal Income Past 12 Months CAD**^c^			
$50,000 or more	69 (37.7)	205 (50.1)	7.84, *p* = .005*
Less than $50,000	114 (62.3)	204 (49.9)	
**Frequency Overwhelmed by Demands in a Typical Week**			
Never or a Few Times	70 (38.3)	343 (83.9)	124.69, *p* < .001*
Often or Most of the Time	113 (61.7)	66 (16.1)	

^a^Generalized Anxiety Disorder Scale (GAD-7) scores dichotomized: *no significant* GAD (GAD-7 < 10 and *probable* GAD (GAD-7 > 9)

^b^Cumulative Lifetime Violence Severity

^c^Canadian Dollars

^*^p < *.05*

### Aim 2. testing the hypothesis that GAD is associated with and predicted by CLV

Unadjusted odds for probable GAD increased by a factor of 10.36 for every increase of 1 in CLV as measured by the CLVS-44 total score (see Table 2). Each CLVS-44 sub-scale significantly increased the odds of experiencing probable GAD; however, the *C8–Lifetime Target Physical Violence from Family Members or Others with Power Over Them* subscale failed to meet the assumption of linearity as assessed with the Box-Tidwell Transformation and was not retained in the multivariable model [52]. The multivariable model containing the 10 remaining CLVS-44 subscales accounted for 23.7% of the variance in GAD. The four subscales that were significantly associated with GAD were retained in the final model which was statistically significant (104.67, *df* = 4, *p* = .000), accounting for 22.8% of the variance in GAD, and correctly classifying 73.8% of the cases. The *C2* subscale included four items about experiences being targeted for physical and psychological violence under the age of 18 years as part of a team or group, and at school, home or in the community from a peer with each increase of 1 raising the odds of probable GAD by a factor of 1.36. C3, a subscale which consisted of 5 items about perpetration of forced sexual activity in childhood and adulthood as part of a team or group, in the community, or in a dating/partner relationship had an *a*OR of 2.70 for GAD. The *a*OR for GAD was 1.57 for C4, a subscale including 5 items about being an adult target of psychological violence at work or in the community including harassment and stalking. The *C7* subscale had 4 items about physical and psychological IPV, two each for childhood and adulthood and raised the odds of probable GAD by a factor of 1.79 for each increase of 1.

**Table 2:** Cumulative Lifetime Violence Severity as Predictor of Generalized Anxiety Disorder Severity^a^ (*N* = 592)

	**Unadjusted Odds Ratios for CLVS **^b^**-44 Total Score and CLVS-44 Subscale Total Scores as Predictors of GAD Severity**				**Adjusted Odds Ratios for CLVS-44 Subscale Scores in a Predictive Model of GAD Severity**			**Final Predictive Model of GAD Severity by CLVS-44 Subscale Scores**		
	***B (SE)***	***OR (95% CI)***	***p***	***Nagelkerke R***^***2***^	***B (SE)***	***aOR (95% CI)***	***p***	***B (SE)***	***aOR (95% CI)***	***p***
**CLVS-44 Total**	2.35 (0.28)	10.36 (6.03, 17.79)	.000*	.218						
**C1-Lifetime Perpetrator Physical & Psychological Violence—Not IPV or Workplace**	1.12 (0.18)	3.12 (2.18, 4.44)	.000*	.10	- 0.21 (0.34)	.81 (0.42, 1.58)	.539			
**C2-Childhood Target Physical & Psychological Peer/Team Violence**	0.63 (0.12)	1.87 (1.49, 2.35)	.000*	.07	0.31 (0.14)	1.36 (1.03, 1.80)	0.28*	0.31 (0.14)	1.36 (1.04, 1.78)	.025*
**C3-Lifetime Perpetrator Sexual Violence**	1.49 (0.24)	4.43 (2.77, 7.10)	.000*	.12	0.88 (0.45)	2.42 (1.01, 5.79)	.047*	0.99 (0.27)	2.70 (1.59, 4.57)	.000*
**C4-Adult Target Psychological Violence—Workplace, Stalking, or Messaging**	0.97 (.14)	2.63 (2.01, 3.43)	.000*	.12	0.47 (0.18)	1.59 (1.11, 2.28)	.011*	0.45 (0.16)	1.57 (1.14, 2.17)	.006*
**C5-Childhood Target Sexual Violence**	0.97 (0.14)	2.64 (2.00, 3.50)	.000*	.11	0.15 (0.20)	1.16 (0.79, 1.71)	.430			
**C6-Adult Target and Perpetrator Violence related to Nature of Work or Civil/Political Unrest**	0.83 (0.16)	2.29 (1.68, 3.12)	.000*	.05	-.26 (0.25)	.77 (.478, 1.25)	.294			
**C7-Lifetime Target Physical & Psychological Dating/Partner Violence**	0.63 (0.12)	1.88 (1.50, 2.35)	.000*	.07	0.54 (0.15)	1.71(1.27, 2.31)	.000*	0.58 (0.15)	1.79 (1.34, 2.38)	.000*
**C-8-Lifetime Target Physical Violence from Family or Others with Power Over Them**	0.88 (.13)	2.41 (1.88, 3.10)	.000*	.12^#^						
**C9-Lifetime Perpetrator Messaging & Stalking**	1.40 (0.21)	4.04 (2.70, 6.05)	.000*	.13	0.49 (0.32)	1.63 (0.87, 3.08)	.124			
**C10-Adult Perpetrator Workplace Psychological & Gender-based (or other Characteristic) Violence**	0.85 (0.16)	2.34 (1.73, 3.18)	.000*	.07	0.20 (0.23)	1.02 (0.65, 1.61)	.931			
**C11-Lifetime Perpetrator Physical Dating/Partner Violence**	0.92 (0.17)	2.50 (1.79, 3.49)	.000*	.07	-0.05 (0.27)	0.95 (0.56, 1.62)	.849			
**Constant**					-4.49 (0.46)	0.01	.000*	-4.47 (0.46)	0.11	.000*
**Model chi-squared**					109.07, *df* = 10, *p* = *.*000*			104.67, *df* = 4. *p* = .000*		
**Nagelkerke R**^**2**^					.237			.228		

^a^Generalized Anxiety Disorder Scale (GAD-7) scores dichotomized: *no significant* GAD (GAD-7 < 10; reference category) and *probable* GAD (GAD-7 > 9)

^b^Cumulative Lifetime Violence Severity-44 Scale

^*^Significant at .05

^#^Violates assumption of linearity; not included in multivariate model

*B* Beta, *SE* Standard Error*, OR* Odds Ratio, *CI* Confidence Interval, *p* probability value, *Nagelkerke R*^*2*^ an approximation of the coefficient of determination, *df* degrees of freedom

### Aim 3. testing the multivariable model of GAD

ORs for each proposed concept in the multivariable theoretical model except Indigeneity were significantly associated with GAD at *p* < 0.1 (see Table 3) and were retained in the model. The adjusted multivariable model was statistically significant, χ^2^ (14) = 268.07, *p* = *.000*, and explained 51.3% of the variance (see Table 4). Significant predictors of GAD were CLV, masculine discrepancy stress, immigration status, current employment, food insecurity, social support, mastery and frequency overwhelmed by demands in a typical week. These variables were retained for the final model and validated using bootstrapping with BCa CIs. The final model was statistically significant, χ^2^ (8) = 264.43, *p =* *.000*, explained 50.8% of the variance in anxiety severity, and correctly classified 83.1% of the cases, 65.6% of cases with probable GAD, and 91% with no significant GAD.

**Table 3:** Descriptive Profile and Unadjusted Odds Ratio for Hypothesized Predictors of Generalized Anxiety Disorder Severity^a^ (*N* = 592)

Model Concepts	Measures	Descriptives	Unadjusted *OR*
**Cumulative Lifetime Violence**	Cumulative Lifetime Violence Severity Total Score	μ (range): 1.53 (1.01 to 3.69)	10.36 (*p* = .000) *
**Gender**	Masculine Discrepancy Stress	μ (range): 15.45 (5 to 35)	1.08 (*p* = .000) *
**Social Location**			
Age	Age in years:	μ (range): 47.41 (19 to 88)	0.97 (*p* = .000) *
Immigration Status		n (%)	
	- Newcomer (*RC*) ^b^ - Born in Canada	121 (20.4) 471 (79.6)	1.54 (*p* = .065) *
Indigenous Canadian		n (%)	
	- Indigenous *(RC)* - Not Indigenous	40 (6.8) 552 (93.2)	0.92 (*p* = .822)
Sexual Orientation		n (%)	
	- Heterosexual only (*RC*) - Gay, Bisexual, another sexual identity	510 (86.1) 82 (13.9)	1.62 (*p* = .050) *
**Socio-economic Disparity**			
Current Employment		n (%)	
	- Employed (*RC*) - Not employed	342 (57.8) 250 (42.2)	1.37 (*p* = .081) *
Financial Strain		n (%)	
	- None to a little (*RC*) - Difficult to impossible to live on income	462 (78.0) 130 (22.0)	5.10 (*p* = .000) *
Food Insecurity		n (%)	
	- Food Secure (*RC)* - Food Insecure	360 (60.8) 232 (39.3)	6.83 (*p* = .000) *
Adverse Housing		n (%)	
	- Stable Housing (*RC*) - Adverse Housing	458 (77.4) 134 (22.6)	5.33 (*p* = .000) *
Personal Income in past 12 Months (CAD) ^c^		n (%)	
	- $50,000 or more (*RC)* - Less than $50,000	274 (46.3) 318 (53.7)	1.67 (*p* = .005) *
**Personal Resources**			
Social Support	Medical Outcomes Social Support Scale—Short Total Score	μ (range): 14.38 (5 to 25)	0.91 (*p* = .000) *
Mastery	Mastery Scale Total	μ (range): 18.51 (7 to 28)	0.81 (*p* = .000)
**Allostatic Overload**			
Overwhelmed by Demands in Typical Week	Frequency overwhelmed by demands in a typical week	n (%)	8.39 (*p* = .000) *
	- Never or a few times (*RC*)	413 (69.8)	
	- Often or most of the time	179 (30.2)	
Chronic Physical Conditions	Number of chronic physical conditions problematic in the past 6 months	μ (range): 1.56 (0 to 9)	1.19 (*p* = .000) *

^a^Generalized Anxiety Disorder Scale (GAD-7) scores dichotomized: *no significant GAD* (GAD-7 < 10; reference category) and *probable GAD* (GAD-7 > 9)

^b^Reference Category

^c^Canadian Dollars

^*^Significant at *p* <  = *.1*

*OR* Odds Ratio, μ mean, *RC* Reference category

**Table 4:** Logistic Regression Model *aORs* for Predictors of Generalized Anxiety Disorder Severity^a^ (*N* = 592)

	**Model of Hypothesized Predictors**			**Final Model**		
	***B (SE)***	***aOR (95% CI)***	***p***	***B (SE)***	***aOR (95% CI)***	***p***
**Lifetime Cumulative Violence:**						
CLVS-44 ^b^ Total Score	1.55 (0.33)	4.70 (2.46, 9.01)	.000*	1.67 (0.32)	5.30 (2.82, 9.94)	.000*
**Gender:**						
Masculine Discrepancy Stress	0.04 (0.02)	1.04 (1.00, 1.07)	.027*	0.04 (0.16)	1.04 (1.01, 1.07)	.013*
**Social Location:**						
Age in Years	- 0.01 (0.01)	0.99 (0.98, 1.01)	.373			
Immigration Status (RC: Newcomer)	0.72 (0.31)	2.04 (1.12, 3.73)	.021*	0.71 (0.30)	2.04 (1.13, 3.70)	.019*
Sexual Orientation (RC: Heterosexual only)	0.19 (0.33)	1.21 (0.63, 2.33)	.573			
**Socio-economic Disparity:**						
Current Employment (RC: Employed)	0.63 (0.29)	1.87 (1.06, 3.30)	.031*	0.59 (0.25)	1.80 (1.12, 2.91)	.016*
Financial Strain (RC: None to a little)	0.11 (0.30)	1.01 (0.56, 1.83)	.972			
Food Insecurity (RC: Food Secure)	0.81 (0.28)	2.25 (1.30, 3.89)	.004*	1.01 (0.24)	2.73 (1.70, 4.39)	.000*
Adverse Housing (RC: Stable Housing)	0.37 (0.30)	1.44 (0.80, 2.58)	.223			
Personal Income Past 12 Months (RC: = > $50,000 CAD)	- 0.01 (0.28)	0.97 (0.57, 1.71)	.974			
**Personal Resources:**						
Social Support	- 0.06 (0.03)	0.94 (0.89, 0.99)	.013*	-0.06 (0.03)	0.94 (0.90, 0.99)	.013*
Mastery	- 0.07 (0.03)	0.93 (0.87, 0.99)	.030*	-0.08 (0.03)	0.93 (0.87, 0.99)	.027*
**Allostatic Overload:**						
Overwhelmed by Demands in Typical Week (RC: Never or a few times)	1.76 (0.27)	5.79 (3.43, 9.76)	.000*	1.83 (0.25)	6.26 (3.82, 10.25)	.000*
Number of Chronic Physical Conditions	0.08 (0.08)	1.07 (0.93, 1.25)	.310			
**Constant**	- 3.43 (1.09)	0.03	.002*	- 3.91 (0.95)	0.02	.000*
**Model Chi-Square**	268.07, *df* = 14, *p* = .000*			264.43, *df* = 8, *p* = .000*		
**Nagelkerke *R***^***2***^	.513			.508		

^a^Generalized Anxiety Disorder Scale (GAD-7) scores dichotomized: *no significant* GAD (GAD-7 < 10 (reference category) and *probable* GAD (GAD-7 > 9)

^b^Cumulative Lifetime Violence Severity-44 Scale

^*^Significant at .05

*B* Beta, *SE* Standard Error*, aOR* Adjusted Odds Ratio, *CI* Confidence Interval, *p* probability value, *RC* Reference category, *df* degrees of freedom*, Nagelkerke R*^*2*^ an approximation of the coefficient of determination

## Discussion

Our study results offer *evidence* that among men, CLV as both target and perpetrator is associated with increased vulnerability to probable GAD. Our finding that certain SDOH in addition to CLV significantly increased the odds of GAD provides guidance about the circumstances of men for whom anxiety may be problematic. Together, these outcomes offer *new* knowledge regarding which chronic stressors and/or social inequities may be of noteworthy concern in the progression of GAD among men [1]. They also have implications for the focus of strength-based trauma- and violence-informed (TVI) health promotion and interventions for boys and men [53, 54].

We identified the prevalence of probable GAD in this *national* sample to be 30.9% in June 2020, a rate higher than the 23.7% we found among 656 Eastern Canadian men with similar CLV histories sampled from 2016 to 2018. This discrepancy may reflect variation in violence exposure and/or recognition of anxiety symptoms by men from different Canadian regions. The prevalence of 30.9% in the current analysis also exceeds that of 20.5% found among men in a crowdsource sample collected by Statistics Canada in April and May of 2020 [55]; yet both samples were collected early in the COVID-19 pandemic. The higher percentage of GAD in our sample, thus, may be partially attributed to factors beyond the pandemic including CLV.

The usefulness of both the CLVS-44 total score and the CLVS-44 sub-scale scores for uncovering the implications of CLV for health, specifically GAD, is demonstrated by this analysis. The CLVS-44 is a comprehensive measure of physical, psychological and sexual violence severity as target and perpetrator, in diverse contexts in childhood and adulthood [20]. Its total score represents *cumulative* violence severity from intersections of multiple, occurring and recurring lifetime violence experiences and overcomes the widespread pitfall of more restricted measures that neglect the effects of other unmeasured lifetime violence. Consequently, measurement with the CLVS-44 adds credibility to our finding that CLV significantly increases the odds of GAD by a factor of 5.30 in the model adjusted for SDOH associated with anxiety in general populations.

Beyond this robust contribution, CLVS-44 sub-scale scores add vital information regarding which unique patterns of CLV are relevant for particular health outcomes [20]. Each CLVS-44 sub-scale includes a subset of items focused on specific types, contexts, or life stages of violence as target and/or perpetrator. Three significant subscales in the adjusted subscale model suggest a continuum of CLV, from being targeted initially in childhood by peers in teams, groups or dating relationships at home, school or in the community, and later in adulthood in partner relationships for physical or psychological violence as well as for psychological violence in the workplace or community that may significantly affect the onset and course of GAD. The fourth significant subscale supported an association between lifetime perpetration of sexual violence and GAD. This new evidence shows that perpetration and victimization may intersect to influence health outcomes such as GAD and highlights the importance of considering men’s experiences of both. Therefore, a starting point for GAD prevention among men with CLV may be TVI support for boys and adolescents fostering constructive relationships and conflict management using strategies that engender trust, augment strengths, and offer choice. Going forward, future research is needed to determine whether these findings are specific only to this national sample of men or can be replicated in diverse samples of men with CLV.

Importantly, our results suggest potentially modifiable social factors that may increase vulnerability for GAD among men with CLV. Our novel finding that the frequency of *feeling overwhelmed or stressed by demands in a typical week* increases odds of GAD more than sixfold in the adjusted multivariable model raises questions about the chronic stress of everyday living among men with CLV. In the study of organized violence in conflict and post-conflict settings, Miller and Rasmussen noted that a substantial proportion of variance in mental health outcomes is unexplained in models using only violence exposure as a predictor; they reported that adding a measure of distress associated with social and material conditions of everyday life increased the explanatory power of models predicting mental health outcomes [38]. Our findings suggest this may also pertain in the study of CLV, implying that the fallout from CLV on the conditions of men’s everyday lives is a discrete chronic stressor, separate from the stress of violence alone, that adds to allostatic overload. The timing of our data collection during the COVID-19 pandemic may have influenced the contribution of distress from these demands to the likelihood of probable GAD. Nonetheless, this result infers the need for TVI interventions for anxiety that include practical strategies to mitigate overwhelming demands of everyday life among men with CLV. As well, future qualitative research is required to better understand the nature of these demands and how they intersect with other SDOH to affect anxiety severity.

Masculine discrepancy stress was also significant in our multivariable model. A strength of the MDS measure is its applicability to men with diverse beliefs about masculine identity, roles, and relationships. It permits men to define their masculinity in terms of their personal perceptions of manliness rather than the prevailing hegemonic indicators of masculinity and captures how much they are bothered by their own and others’ perceptions of their masculinity [23]. Because not all men who feel they do not measure up to standards of masculinity will be distressed, this finding offers a means of distinguishing which men with CLV may be more vulnerable to anxiety. The connection between higher MDS and greater likelihood of GAD among men with histories of violence, to our knowledge, has not been previously reported.

We also considered the effects of diversity in social location (i.e., age, immigration status, Indigeneity, sexual orientation). Due to an insignificant (> .1) unadjusted association with anxiety severity, Indigeneity was not included in the multivariable LR. This exploratory study was not designed to meet the criteria for Indigenous Health Research [56] and the sample of participants who self-identified as Indigenous may have been too small to achieve significance. In the multivariable model, the only significant indicator of social location was immigration status. Our finding that men born in Canada were twice as likely to have probable GAD than those who were newcomers is similar to that found for people born in Canada compared to immigrants [6]. Migrants in Canada for less than 10 years have been found to have lower rates of anxiety disorders than those in Canada 10 years and longer [57]. In our sample, most (85.1%) of newcomers reported living in Canada for more than 5 years and less than 2% of men were identified as refugees. Interestingly, in a recent systematic review, although rates of PTSD and depression were higher among refugees and asylum seekers, rates of anxiety were similar to that of general populations [58]. In future research, specific data on number of years living in Canada and the inclusion of more immigrants who came to Canada as refugees or asylum seekers would permit a more nuanced exploration of how the nature of immigration influences the likelihood of GAD in men with CLV. Our focus on diversity was also limited by our inability to include data on ethnicity in the analysis. Overall, men identified with more than 20 different ethnicities; individual men identified with 1 to 7 with the majority (82.1%) reporting only one.

Our model shows that men are affected simultaneously by multiple SDOH and supports the assertion that the nexus among SDOH for individuals may lead to health advantages or disparities [59]. With respect to socio-economic disparity, our finding that unemployed men with CLV were almost twice as likely than employed men to experience probable GAD is new knowledge. Although unemployment among men has been found to more than double the likelihood of anxiety [34], and also to be associated with adverse childhood experiences including physical, sexual and verbal abuse as well as witnessing domestic violence [60], we located no studies that linked lifetime violence with *both* unemployment and anxiety severity. Our data were collected just after approximately 15% of Canadian men experienced job loss due to COVID-19 [61]. Because loss of income has been associated with mental illness [36], our finding may be, in part, specific to the pandemic. This is reinforced by the rate of unemployment being 31.5% among eastern Canadian men with CLVS histories in our previous study as compared 42.2% in the present study. Another indicator of socio-economic disparity found to significantly increase anxiety severity was food insecurity (*a*OR = 2.25). In Canada, 12.8% of households experienced food insecurity in 2017/18 [62], a rate that increased to 14.6% in May 2020 during the pandemic [63]. Based on our data collected in June 2020, 39.3% of men reported food insecurity. About 25% of those experiencing job insecurity associated with COVID-19 in Canada also experienced food insecurity [64], suggesting that anxiety levels in our sample may be associated with intersections among CLVS, unemployment and food insecurity.

Limited research exists about factors that reduce the likelihood of anxiety among men. Therefore, our result regarding possible protection offered by personal resources against anxiety is of note. Each increase of 1 in the social support total score decreased men’s likelihood of GAD by 6% (*a*OR = 0.94). To our knowledge this is the first evidence in a sample of men experiencing violence that social support is associated with lower odds of GAD, although among women with IPV, this relationship has been significant [65, 66]. We also found that each increase of 1 in mastery total score reduced the odds of GAD by 7% (*a*OR = 0.93) in our sample, a finding that is consistent with that of Bebanic et al. [27] who found that mastery partly mediated the association between comorbid anxiety and depression in *men* and past year physical and psychological violence as target. No other studies were located to reinforce this relationship. Overall, our finding that social support and mastery may play a role in reducing likelihood of GAD in men experiencing CLVS underscores possibilities for strength-building interventions to mitigate anxiety.

Because the CLVS-44 measures violence in childhood and in adulthood as *both* target and perpetrator, our results support a common etiology underpinning both violence perpetration and victimization [67]. Recognition of men’s possible positions as both victim and perpetrator [68] is foundational in TVI care for anxiety that focuses on choice, safety, trust, and shared decision-making [53, 54]. Such interventions play a role in reducing future violence perpetration by men who address detachment from CLV by exerting power and control in periods of high distress and anxiety to gain self-worth and connect with others [28]. Thus, it is important for health care providers to consider the impact of both past and ongoing violence on the progression of anxiety, and to resist assumptions that violence is merely a reflection of ‘being a man’.

### Limitations

This cross-sectional analysis offers support for relationships among variables but these associations are not causal. Importantly, the findings are new and have potential to inform the design of future longitudinal research to better understand how CLVS and SDOH intersect to affect GAD overtime. Our methods captured general associations for the whole sample, but neglect the heterogeneity of experiences among men in the sample. Going forward, stratification of analysis by variables such as age group, food insecurity and employment status or use of variable-oriented methods such as latent class analysis may better illuminate distinct sub-groups of men who may be most vulnerable to GAD. The study is also limited by the predominantly heteronormative perspective of the literature used to develop the model; thus, findings may not be applicable to trans men or those who do not identify as heterosexual.

## Conclusion

In summary, we conclude that Canadian men with lifetime violence histories are a sub-group disproportionately affected by chronic stressors including CLVS as well as socio-economic disparities that may increase vulnerability for probable GAD. We found the prevalence of probable GAD among Canadian men with histories of lifetime violence to be higher than rates within the general population even when the confounding effects of COVID-19 were considered. Our final eight-variable model is striking in that it accounts 50.8% of the variance in GAD severity, revealing the simultaneous contributions of CLVS, feeling overwhelmed by daily stressors, masculine discrepancy stress, immigration status, unemployment and food insecurity. But importantly, the model also demonstrates the possible protective effects of both social support and mastery, as well as socio-economic predictors such as food insecurity and unemployment that are amenable to change at community and structural levels. Our model provides provisional guidance for identifying which men with CLVS might benefit from early diagnosis and treatment to prevent years of disability associated with GAD [3].

## Data Availability

The dataset analysed in this study is not publicly available because permission was not obtained from participants for deposition in a public repository but the dataset is available from Dr. Kelly Scott-Storey kscottst@unb.ca on reasonable request.

## Abbreviations

*a*OR: Adjusted odds ratios
BCa: Bias-corrected and accelerated
CAD: Canadian dollars
CI: Confidence intervals
CLV: Cumulative lifetime violence
CLVS: Cumulative lifetime violence severity
CLVS-44: Cumulative Lifetime Violence Severity Scale-44
GAD: Generalized Anxiety Disorder
GAD-7: Generalized Anxiety Disorder scale
IPV: Intimate partner violence
LR: Logistic regression
MDS: Masculine discrepancy stress
OR: Odds ratio
PTSD: Posttraumatic stress disorder
SDOH: Social determinants of health

## References

[1] Zvolensky M, Garey L, Bakhshaie J (2017). Disparities in anxiety and its disorders. J Anxiety Disord.

[2] GBD 2016 Disease Injury and Incidence and Prevalence Collaborators. Global, regional, and national incidence, prevalence, and years lived with disability for 328 diseases and injuries for 195 countries, 1990–2016: A systematic analysis for the Global Burden of Disease Study 2016. The Lancet. 2017;390:1211–1259. 10.1016/S0140-6736(17)32154-2 10.1016/S0140-6736(17)32154-2 PMC5605509 28919117

[3] Stein D, Scott K, de Jonge P, Kessler R (2017). Epidemiology of anxiety disorders: from surveys to nosology and back. Dialogues Clin Neurosci.

[4] Silva M, Loureiro A, Cardoso G (2016). Social determinants of mental health: a review of the evidence. Eur J Psychiatry.

[5] Smith D, Mouzon D, Elliot M (2018). Reviewing assumptions about men’s mental health: an exploration of the gender binary. Am J Men’s Health.

[6] Watterson R, William, Lavorato D, Patten S. Descriptive epidemiology of generalized anxiety disorder in Canada. Can J Psychiatry. 2017;26:24–29. 10.1177/0706743716645304 10.1177/0706743716645304 PMC5302105 27310239

[7] Haegerich T, Hall J (2011). Violence and men’s health: Understanding the etiological underpinnings of men’s experiences with interpersonal violence. Am J Lifestyle Med.

[8] McEwen B (2017). Neurobiological and systemic effects of chronic stress. Chronic Stress.

[9] Harvard Medical School. 12-month prevalence of DSM-IV/WMH-CIDI disorders by sex and cohort. https://www.hcp.med.harvard.edu/ncs//ftpdir/table_ncsr_12monthprevgenderxage.pdf Accessed 20 May 2021.

[10] Kusminskaite E, Penninx B, van Harmelen A, Elzinga B, Hovens J, Vinkers C (2021). Childhood trauma in adult depressive and anxiety disorders: an integrated review on psychological and biological mechanisms in the NESDA cohort. J Affect Disord.

[11] Turner S, Taillieu T, Cheung K, Afifi T (2017). The relationship between child sexual abuse and mental health outcomes among males: results from a nationally representative Unites States sample. Child Abuse Negl.

[12] Próspero M (2007). Mental health symptoms among male victims of partner violence. Am J Men’s Health.

[13] Spencer C, Mallory A, Cafferky B, Kimmes J, Beck A, Stith S (2019). Mental health factors and intimate partner violence perpetration and victimization: a meta-analysis. Psychol Violence.

[14] Choudhary E, Smith M, Bossarte R (2012). Depression, anxiety, and symptom profiles among female and male victims of sexual violence. Am J Men’s Health.

[15] Lee J (2021). Pathways from childhood bullying victimization to young adult depressive and anxiety symptoms. Child Psychiatry Hum Dev.

[16] O’Donnell S, MacIntosh J (2016). Gender and workplace bullying: men’s experiences of surviving bullying at work. Qual Health Res.

[17] Coid J, Ulrich S, Keers R, Bebbington P, DeStavola B, Kallis C, Yang M, Reiss D, Jenkins R, Donnelly P (2013). Gang membership, violence and psychiatric morbidity. Am J Psychiatry.

[18] Mngoma N, Fergus S, Jeeves A, Jolly R (2016). Psychosocial risk and protective factors associated with perpetration of gender-based violence in a community sample of men rural KwaZulu-Natal. S Afr Med J.

[19] Scott-Storey K (2011). Cumulative abuse: do things add up? An evaluation of the conceptualization, operationalization, and methodological approaches in the study of the phenomenon of cumulative abuse. Trauma Violence Abuse.

[20] Scott-Storey K, O’Donnell S, Wuest J, MacIntosh J, Merritt-Gray M (2020). Cumulative lifetime violence severity scale: development and initial testing among men. BMC Public Health.

[21] World Health Organization: Global status report on violence prevention 2014. https://www.who.int/publications/i/item/9789241564793 (2014). Accessed 12 March 2017.

[22] Courtenay W (2000). Constructions of masculinity and their influence on men’s well-being: a theory of gender and health. Soc Sci Med.

[23] Reidy D, Brookmeyer K, Gentile B, Berke D, Zeichner A (2016). Gender role discrepancy stress, high-risk sexual behavior and sexually transmitted disease. Arch Sex Beh.

[24] Reidy D, Smith-Darden J, Vivolo-Kantor A, Malone C, Kernsmith P (2018). Masculine discrepancy stress and psychosocial maladjustment: implications for behavioral and mental health of adolescent boys. Psychol Men Masc.

[25] Drioli-Phillips P, Oxlad M, Feo R, Scholz B, LeCouteur A (2020). “I feel abused by my own mind”: Themes of control in men’s online accounts of living with anxiety. Qual Health Res.

[26] Pearlin L, Bierman A, Aneshensel C, Phelan J, Bierman A (2013). Current issues and future directions in research into the stress process. Handbook of the sociology of mental health.

[27] Bebanic V, Clench-Aas J, Raanaas R, Nes R (2017). The relationship between violence and psychological distress among men and women: do sense of mastery and social support matter?. J Interpers Violence.

[28] Taylor P, O’Donnell S, Wuest J, Scott-Storey K, Vincent C, Malcolm J (2021). The mental health effects of cumulative lifetime violence in men: disruptions in the capacity to connect with others and finding ways to reengage. Glob Qual Health Res.

[29] Kavanagh S, Graham M. How gender inequity impacts on men’s health. An exploration of theoretical pathways. IJMSCH. 2019;2:e11-e2.1. 10.22374/ijmsch.v2i1.5

[30] Arenas D, Thomas A, Wang J, DeLisser H (2019). A systematic review and meta-analysis of depression, anxiety, and sleep disorders in US adults with food insecurity. J Gen Intern Med.

[31] Jessiman-Perreault G, McIntyre L.The household food insecurity gradient and potential reductions in adverse population mental health outcomes in Canadian adults. SSM – Population Health. 2017;3:464–472. 10.1016/j.ssmph.2017.05.013 10.1016/j.ssmph.2017.05.013 PMC5769073 29349239

[32] Glasheen C, Forman-Hoffman V, Heddon S, Ridenour T, Wang J, Porter J (2019). Residential transience among adults: Prevalence, characteristics, and association with mental illness and mental health service use. Community Ment Health J.

[33] Vásquez-Vera H, Palència L, Magna I, Neira J, Borrell C (2017). The threat of home eviction and its effects on health through an equity lens: a systematic review. Soc Sci Med.

[34] Acevedo P, Mora-Urda A, Montero P (2019). Social inequalities in health; duration of unemployment unevenly effects on the health of men and women. Eur J Public Health.

[35] Dobson K, Vigod S, Mustard C, Smith P. Trends in the prevalence of depression and anxiety disorders among working-age Canadian adults between 2000 and 2016. Health Reps. 2020;3:12–23. 10.25318/82‐003‐x202001200002‐eng 10.25318/82-003-x202001200002-eng 33325673

[36] Ridley M, Rao G, Schilbach F, Patel V. Poverty, depression, and anxiety: Causal evidence, and mechanisms. Science. 2020;370. 10.1126/science.aay0214 10.1126/science.aay0214 33303583

[37] Dijkstra-Kersten S, Biesheuvel-Leliefeld K, van der Wouden J, Penninx B, van Marwijk H (2015). Associations of financial strain and income with depressive and anxiety disorders. J Epidemiol Community Health.

[38] Miller K, Rasmussen A (2010). War exposure, daily stressors, and mental health in conflict and post-conflict settings: Bridging the divide between trauma-focused and psychosocial frameworks. Soc Sci Med.

[39] Gilmour H. Sexual orientation and complete mental health. Health Reps. 2019;30:3–10. 10.25318/82-003-x201901100001-eng 10.25318/82-003-x201901100001-eng 31747043

[40] Gopalkrishnan N (2018). Cultural diversity and mental health: considerations for policy and practice. Front Public Health.

[41] Scott K, Lim C, Al-Hamzawi,A, Alonso J, Bruffaerts R, Caladas-de-Almeida J, Florescu,S, …Kessler R. Association of mental disorder with subsequent chronic physical conditions: World mental health surveys from 17 countries. JAMA Psychiatry. 2016;73:150–158. 10.1001/jamapsychiatry.2015.2688 10.1001/jamapsychiatry.2015.2688 PMC5333921 26719969

[42] Reidy D, Berke D, Gentile B, Zeichner A (2014). Man enough? masculine discrepancy stress and intimate partner violence. Personality Individ Differ.

[43] Ali J, Avison W (1997). Employment transitions and psychological distress: the contrasting experiences of single and married mothers. J Health Soc Behav.

[44] Gunderson C, Engelhard E, Crumbaugh A, Seligman H (2017). Brief assessment of food insecurity accurately identifies high-risk US adults. Public Health Nutr.

[45] Sandel M, Sheward R, Ettinger de Cuba S, Coleman S, Frank D, Chilton M, Black M, Heeren T, PasQuariello J, Casey P, Ochoa E, Cutts D.Unstable housing and caregiver and child health in rental families. Pediatrics. 2018;141. http://pediatrics.aappublications.org/content/141/2/e20172199 10.1542/peds.2017-2199 29358482

[46] McCarrier K, Bushnell D, Martin M, Paczkowski R, Nelson D, Buesching D. Validation and psychometric evaluation of a 5-item measure of perceived social support. Value in Health. 2011;14. https://www.ispor.org/heor-resources/presentations-database/presentation/ispor-16th-annual-international-meeting/validation-and-psychometric-evaluation-of-a-5-item-measure-of-perceived-social-support

[47] Pearlin L, Lieberman M, Menaghan E,Mullan, J. The stress process. J Health Soc Behav.1981;22:337–356. https://www.jstor.org/stable/2136676 7320473

[48] Spitzer R, Kroenke K, Williams J, Lowe, B. A brief measure for assessing generalized anxiety disorder. The GAD-7. Arch Intern Med. 2006;166:1092–1097. 10.1001/archinte.166.10.1092 10.1001/archinte.166.10.1092 16717171

[49] Löwe B, Decker O, Müller S, Brähler E, Schellberg D, Herzog W, Herzberg P (2008). Validation and standardization of the generalized anxiety disorder screener (GAD-7) in the general population. Med Care.

[50] Ranganathan P, Pramesh C, Aggarwal R (2017). Common pitfalls in statistical analysis: Logistic regression. Perspect Clin Res.

[51] Bujang M, Sa’at N, Sidik T, Lim C. Sample size guidelines for logistic regression from observational studies with large population: Emphasis on the accuracy between statistics and parameters based on real life clinical data. Malays J Med Sci. 2018;25:122–30. 10.21315/mjms2018.25.4.12 10.21315/mjms2018.25.4.12 PMC6422534 30914854

[52] Tabachnick B, Fidell L (2007). Using multivariate statistics.

[53] Fallot R, Bebout R, Poole N, Greaves L (2012). Acknowledging and embracing “the boy inside the man”: Trauma-informed work with men. Becoming trauma-informed.

[54] Ponic P, Varcoe C, Smutylo T. Trauma- (and violence-) informed approaches to supporting victims of violence: Policy and practice considerations. Victims of Crime Research Digest. 2016; 9. https://www.justice.gc.ca/eng/rp-pr/cj-jp/victim/rd9-rr9/p2.html

[55] Moyser M. Gender differences in mental health during the Covid-19 pandemic. StatCan Covid 19, 2020. https://www150.statcan.gc.ca/n1/en/pub/45-28-0001/2020001/article/00047-eng.pdf?st=dq3kARbs

[56] CIHR: Defining indigenous research. https://cihr-irsc.gc.ca/e/50340.html 2021. Accessed 6 Sept 2021.

[57] Edwards J, Hu M, Thind A, Stranges S, Chiu M, Anderson K (2019). Gaps in understanding the epidemiology of mood and anxiety disorders among migrant groups in Canada: a systematic review. Can J Psychiatry.

[58] R Blackmore J Boyle M Fazel S Ranashinha K Gray G Fitzgerald M Misso M Gibson-Helm. 2020. The prevalence of mental illness in refugees and asylum seekers: a systematic review and meta-analysis. PLoS. 10.1371/journal.pmed.1003337 10.1371/journal.pmed.1003337 PMC7505461 32956381

[59] Hankivsky O, Christoffersen A (2008). Intersectionality and the determinants of health: a Canadian perspective. Crit Public Health.

[60] Liu Y, Croft J, Chapman D, Perry G, Greenlund K, Zhao G, Edwards V (2013). Relationship between adverse childhood experiences and unemployment among adults from five US states. Soc Psychiatry Psychiatr Epidemiol.

[61] Lemieux T, Milligan K, Schirle T, Skuterud M. Initial impacts of the COVID-19 pandemic on the Canadian labour market. Can Public Policy. 2020;July:S56 -S64. Doi:10.3138/cpp.2020-049 10.3138/cpp.2020-049 PMC7977000 38629977

[62] Statistics Canada. Household Food Insecurity, 2017/18. Health Fact Sheets. https://www150.statcan.gc.ca/n1/en/pub/82-625-x/2020001/article/00001-eng.pdf?st=FSVl1MEP (2020). Accessed 6 Sept 2021.

[63] Polsky J, Gilmour, H. Food insecurity and mental health during the COVID-19 pandemic. Health Reps. 2020;31:1–11. 10.25318/82‐003‐x202001200001‐eng 10.25318/82-003-x202001200001-eng 33325672

[64] Men F, Tarusuk V. Food insecurity amid the COVID-19 pandemic: Food charity, government assistance, and employment. Can Public Policy. 2021;June:203–230. 10.3138/cpp.2021-001 10.3138/cpp.2021-001 PMC9395161 36039314

[65] Choi A, Lo B, Lo R. Intimate partner violence victimization, social support, and resilience: Effects on the anxiety levels of young mothers. Journal of Interpersonal Violence. 2019. 10.1177/0886260519888532 10.1177/0886260519888532 31789087

[66] Coker A, Smith P, Thompson M, McKeown R, Bethea L, Davis K. Social support protects against the negative effects of partner violence on mental health. J Women’s Health Gender-based Med. 2004;11:465–476.10.1089/15246090260137644 10.1089/15246090260137644 12165164

[67] Hamby S, Grych J (2013). The web of violence: Exploring connections among different forms of interpersonal violence and abuse.

[68] Beresford H, Wood J (2016). Patients or perpetrators? The effects of trauma exposure on gang members’ mental health: a review of the literature. J Criminol Res Policy Pract.

